# Stability, Entrapment and Variant Formation of *Salmonella* Genomic Island 1

**DOI:** 10.1371/journal.pone.0032497

**Published:** 2012-02-23

**Authors:** János Kiss, Béla Nagy, Ferenc Olasz

**Affiliations:** 1 Agricultural Biotechnology Center, Gödöllő, Hungary; 2 Veterinary Medical Research Institute of Hungarian Academy of Sciences, Budapest, Hungary; University of Hyderabad, India

## Abstract

**Background:**

The *Salmonella* genomic island 1 (SGI1) is a 42.4 kb integrative mobilizable element containing several antibiotic resistance determinants embedded in a complex integron segment In104. The numerous SGI1 variants identified so far, differ mainly in this segment and the explanations of their emergence were mostly based on comparative structure analyses. Here we provide experimental studies on the stability, entrapment and variant formation of this peculiar gene cluster originally found in *S*. Typhimurium.

**Methodology/Principal Findings:**

Segregation and conjugation tests and various molecular techniques were used to detect the emerging SGI1 variants in *Salmonella* populations of 17 *Salmonella enterica* serovar Typhimurium DT104 isolates from Hungary. The SGI1s in these isolates proved to be fully competent in excision, conjugal transfer by the IncA/C helper plasmid R55, and integration into the *E. coli* chromosome. A trap vector has been constructed and successfully applied to capture the island on a plasmid. Monitoring of segregation of SGI1 indicated high stability of the island. SGI1-free segregants did not accumulate during long-term propagation, but several SGI1 variants could be obtained. Most of them appeared to be identical to SGI1-B and SGI1-C, but two new variants caused by deletions via a short-homology-dependent recombination process have also been detected. We have also noticed that the presence of the conjugation helper plasmid increased the formation of these deletion variants considerably.

**Conclusions/Significance:**

Despite that excision of SGI1 from the chromosome was proven in SGI1^+^ *Salmonella* populations, its complete loss could not be observed. On the other hand, we demonstrated that several variants, among them two newly identified ones, arose with detectable frequencies in these populations in a short timescale and their formation was promoted by the helper plasmid. This reflects that IncA/C helper plasmids are not only involved in the horizontal spreading of SGI1, but may also contribute to its evolution.

## Introduction

**Figure 1:**
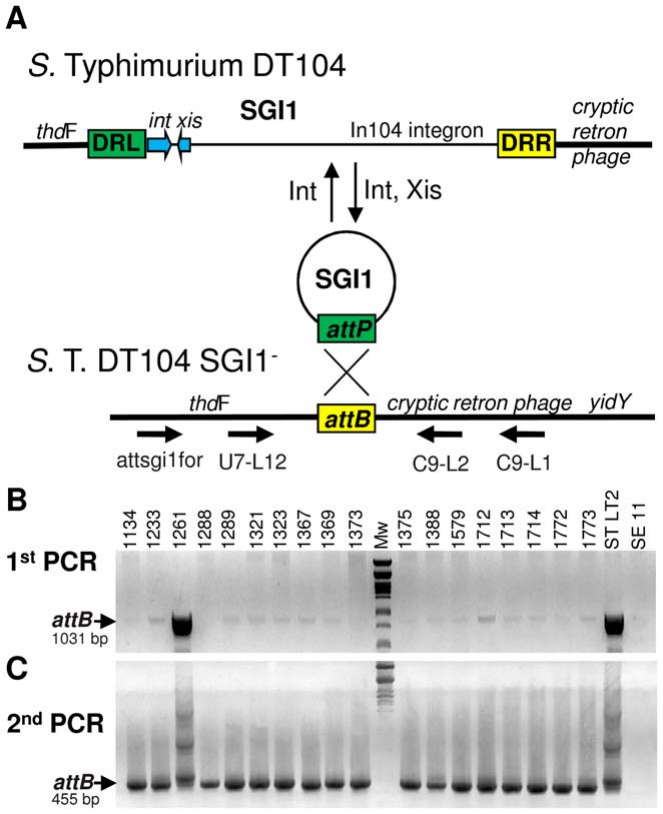
Detection of the spontaneous excision of SGI1 in the Hungarian *S.* T. DT104 isolates. (**A**) Simple representation of the excision/integration cycle of SGI1 on the *S.* T. chromosome. Thick and thin lines represent the *Salmonella* chromosome and SGI1 DNA, respectively. In SGI1, only the elements of the site-specific recombination system are detailed (blue arrows). Green and yellow colours of rectangles representing the recombinogenic sites refer to the sequence identities (DRL/*attP* and DRR/*attB*). The primers applied (Table S1) in the nested PCR for detection of the *attB* site in the SGI1^+^ *S.* T. strains are indicated. (**B**) The panel shows the PCR products obtained from the total DNA of 18 *S.* T. DT104 isolates using primers attsgi1for and C9-L1. The ST LT2 (*Salmonella enterica* serovar Typhimurium LT2 strain MA1703) was an SGI1^−^ positive control and SE11 (*Salmonella enterica* serovar Enteritidis strain 11) devoid of the retron phage served as a negative control. Mw: λ DNA digested with *Pst*I was used as molecular weight standard on each figure. The 1031 bp *attB* specific amplicon is indicated. Note that *S.* T. 1261 showing strong *attB* signal proved to be SGI1-free where the *attB* site is intact. (**C**) The panel shows the products of the second PCR using primers U7-L12 and C9-L2. The 455 bp *attB* specific amplicon is indicated. For PCR parameters see Materials and Methods.

**Figure 2:**
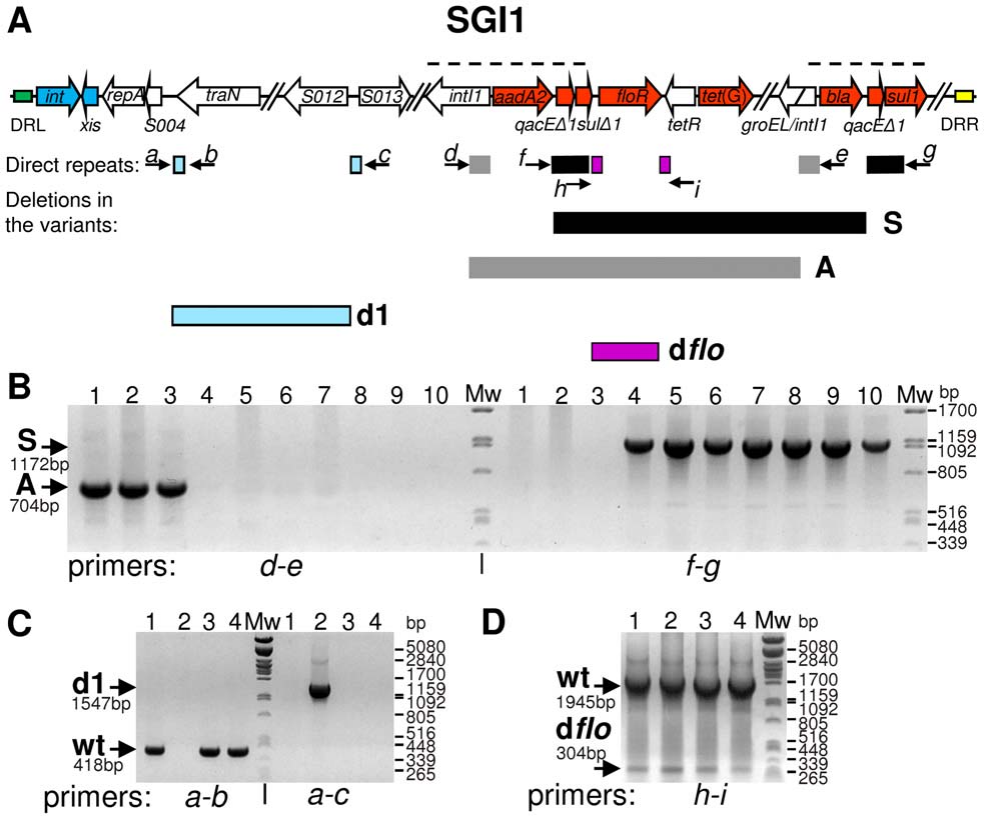
SGI1 deletion variants isolated from the wt form. (**A**) Schematic map of SGI1 (not in scale). ORFs are represented by arrows, resistance genes are red. DRs are shown as green and yellow rectangles. Dashed lines above the graph show the two *IntI1* integron regions. Below the graph, the directly repeated homologous tracts are shown as bars (from left to right): light blue bars: 3588–3618 bp and 13516–13546 bp, dark grey bars: 27552–27992 bp and 36824–37264 bp, black bars: 28840–29822 bp and 38300–39282 bp, purple bars: 30150–30252 bp (*flo*DRL) and 31791–31893 bp (*flo*DRR). Coordinates are according to the annotated SGI1 sequence (GenBank AF261825.2). The lettered arrows show PCR primers used for the amplification of the deletion forms (for primer sequences see Table S1). Long horizontal bars below the graph represent the deleted regions between the direct repeats. Filling of bars corresponds to those of the respective direct repeats. (**B**) PCR test of the isolated A- and S-type deletion variants. The expected size of PCR products is indicated. Lanes 1–10: PCR products from total DNA of strains ST11A/2, ST14A, ST21A, ST11S/1, ST15S, ST18S, ST19S, ST20S, ST21S/1 and ST28S/1. Used primers are indicated below the picture. (**C**) PCR test of the **d1** deletion in the SGI1 variants entrapped in pJKI666. The size of the wt (primers: *a–b*) and the **d1** (primers: *a–c*) amplicon is indicated. Lanes 1–4: PCR products from plasmid DNA of entrapped SGI1 derived from strains ST1773, ST28S/1, ST21S/1 and ST21A. Primers used are indicated below the picture. (**D**) PCR test for the presence of **d** ***flo*** SGI1 deletion variant. The wt and **d** ***flo*** PCR products (primers: *h–i*) and their size are indicated. Lanes 1–2: PCR products from total DNA of ST1375/IP40a, lanes 3–4: ST1375 (2 parallel colonies).

*Salmonella* is one of the most prevalent food-borne zoonotic pathogen. Among more than 2500 serotypes, the majority of infections in humans are caused only by a few serotypes, such as *S. enterica* serovar Enteritidis and Typhimurium. Since the early 1990 s, the spread of a multidrug resistant (MDR) clone of *S*. Typhimurium (*S*. T.) DT104 has been observed among human and domestic animals characterized by resistance to ampicillin, chloramphenicol/florfenicol, streptomycin/spectinomycin, sulphonamides and tetracycline (often designated as ACSSuT) [1]. The region responsible for the MDR phenotype is located on a chromosomal island named SGI1 [2]. The 42.4 kb island (GenBank AF261825.2) contains a complex class 1 integron, In104, related to the In4 group [3], including determinants for the above resistances (Figs 1A and 2A). The 13 kb In104 gene cluster appears to be a transposable unit delimited by the 25 bp inverted repeats IRi and IRt [2]. The facts that these IRs are surrounded by 5 bp direct repeats and a similar gene cluster is located at a different position in an SGI1-related island, SGI2, also suggest the insertional acquisition of In104 segment into the SGI1 backbone [2], [4]. The In104 cluster contains two incomplete integrons with functional *attI1* sites (Fig. 2A). The left integron located near the IRi has an intact 5′ conserved sequence (5′-CS) with a functional *intI1*gene and an *aadA2* gene cassette conferring resistance to streptomycin and spectinomycin (Str^R^ and Spt^R^) in the *attI1* site and an incomplete 3′-CS with the *qacEΔ1sulΔ1* gene fusion. The right integron has a truncated 5′-CS including the *groEL-intI1* fusion with the gene cassette *bla_PSE-1_* conferring resistance to β-lactams (Amp^R^) in the *attI1* site and an intact 3′-CS with *qacEΔ1sul1* fusion conferring resistance to sulphonamides (Sul^R^). The other two resistance genes *floR* and *tet*(G) conferring resistance to chloramphenicol/florfenicol (Chm^R^/Flo^R^) and tetracycline (Tet^R^) are not parts of the integrons. They are located together with a mobile element IS*CR3* [5] in the segment defined by the two integron regions.

Since the discovery of the SGI1 prototype [6], numerous variants designated from SGI1-A to V have been described from several *Salmonella* serotypes and recently from *Proteus mirabilis* strains [3], [7]–[18]. Most of the variants differ in the MDR region, with the exception of an SGI1 isolate from *S. e.* serovar Kentucky, where IS*Vch4* mediated deletion occurred in the SGI1 backbone near to the 5′ end [13], and the SGI1-V, where 2.3 kb insertion occurred in ORF S014 [17]. Involvement of several recombination processes have been presumed in the formation of different MDR regions. Some variants seem to be deletion derivatives of the prototype SGI1 (SGI1-B and C) and that of SGI1-I (SGI1-O), which were possibly generated by homologous recombination, while SGI1-A, D and G are probably derived by the insertion of a putative mobile element, IS*CR1*, into SGI1, SGI1-C and B, respectively. Another class of variants is where antibiotic resistance cassettes vary in the integrons (SGI1-F, H, I, L, M). In these cases, homologous recombination between SGI1 In104 and related integrons located on plasmids or the chromosome has been proposed as the mechanism of incorporation of the variant MDR region into SGI1. Further variants have probably been formed by transposon/IS mediated rearrangements (SGI1-E, K2-5, P1-2, Q1-3). On the other hand, the most complex MDR region found in SGI1-K1, shows a highly mosaic structure that can hardly be deduced from the prototype SGI1, as well as SGI1-V, which also contains a new complex integron region [13], [17].

SGI1 and its variants are located on the chromosome at the 3′ end of *thd*F gene and are delimited by 18 bp imperfect direct repeats (DRL and DRR). DRR corresponds to the last 18 bp of *thd*F (*attB*), while DRL probably derives from the joined ends of the free circular form of SGI1 (*attP*) [19]. The site-specific excision and integration of SGI1 are catalysed by the lambda integrase family member Int and Xis encoded near the 5′ end of the island [19]. Although the SGI1 backbone encodes several conjugation-related genes, its self-transfer could not be observed, but the mobilization of the island by the IncA/C plasmid R55 has been reported [2], [19]. A recent study demonstrated that plasmids belonging to other incompatibility groups cannot mobilize SGI1, but numerous members of IncA/C group are able to help the SGI1 transfer with different efficiencies [20]. After conjugal transfer, SGI1 integrates into the *attB* site of the recipient, however, secondary insertion sites can also be targeted with lower frequencies [21].

In this work we performed experimental studies on the mobility functions and stability of SGI1 in 17 *S.* T. DT104 strains isolated from different animal or food sources in Hungary. Furthermore we aimed to study the molecular basis of variant formation of this genomic island. The strains have earlier been characterised by phage typing and PFGE, showing at least 7 different genotypes among the isolates [22]. Here we demonstrated that SGI1 is able to excise spontaneously from the chromosome in all of these strains, and we could detect a high frequency of SGI1 transfer and integration into the *E. coli* chromosome. Additionally, we constructed a trap vector applicable for the capture of the island via its conjugal transfer. Although the excision ability can theoretically lead to the loss of the island, SGI1-free segregants could not be detected during long term propagation, in turn, segregants with altered resistance phenotype emerged in several cultures. These variants appeared to be identical to SGI1-B and SGI1-C. Additionally, a new variant with a ca. 10 kb deletion between the short direct repeats in the SGI1 backbone was isolated and the appearance of a further deletion variant lacking the *flo*R gene was also confirmed. We concluded that SGI1^+^ strains continuously produce these variants and showed that the presence of conjugation helper plasmids significantly increase their formation. Several hypotheses for the mechanisms are also discussed.

## Results

### Mobility functions of SGI1 in the Hungarian S. Typhimurium isolates

Eighteen florfenicol resistant *S.* Typhimurium DT104 isolates were tested for their antibiotic resistance and the occurrence of SGI1. All but one of the 18 *S.* T. strains showed all the SGI1 related resistance markers and gave positive signals in PCRs specific for the left and right chromosomal junctions DRL and DRR [22]. Furthermore, all the 18 strains proved to be resistant for rifampicin, four for nalidixic acid (ST1579, 1712, 1713 and 1714) and one for gentamicin (ST1388) (Table 1). To test whether the 17 SGI1^+^ strains harbour the fully active SGI1 prototype [19], the excision and the mobilization of the island were examined.

**Table 1:** Bacterial strains used in the experiments.

*Strains*	*Genotype*	*Reference*
*E. coli*		
Ec/R55	*E. coli* strain containing the IncA/C resistance plasmid R55	A. Cloeckaert, p.c.
Ec/IP40a	*E. coli* strain containing the IncA/C resistance plasmid IP40a	B. Doublet, p.c.
TG1	*supE hsd*Δ*5 thi*Δ(*lac-pro*AB) F′[*traD*36 *proAB^+^ lacI* ^q^ *lacZ*ΔM15]	[35]
TG1Nal	Nal^R^ derivative of TG1	This work
TG2	*supE hsd*Δ*5 thi*Δ(*lac-pro*AB)Δ(*srl-rec*A)306::Tn*10*(Tet^R^) F′[*traD*36 *proAB^+^ lacI* ^q^ *lacZ*ΔM15]	[37]
TG90	*pcn* B80 *zad*::*Tn*10 (Tet^R^) derivative of TG1	[36]
TG90Nal	Nal^R^ derivative of TG90	This work
*S*. *e*. Typhimurium.		
LT2 MA1703	*rec*A1, *srl*	L. Bossi, unpublished
ST1134	wt, SGI1^+^, Amp^R^,Chm^R^,Flo^R^,Str^R^,Spt^R^,Sul^R^,Tet^R^,Rif^R^,Kan^S^,Gen^S^,Nal^S^	[22]
ST1233	wt, SGI1^+^, Amp^R^,Chm^R^,Flo^R^,Str^R^,Spt^R^,Sul^R^,Tet^R^,Rif^R^,Kan^S^,Gen^S^,Nal^S^	[22]
ST1261	wt, SGI1^−^, Amp^R^,Chm^S^,Flo^R^,Str^R^,Spt^R^,Sul^S^,Tet^S^,Rif^R^,Kan^S^,Gen^S^,Nal^S^	[22]
ST1288	wt, SGI1^+^, Amp^R^,Chm^R^,Flo^R^,Str^R^,Spt^R^,Sul^R^,Tet^R^,Rif^R^,Kan^S^,Gen^S^,Nal^S^	[22]
ST1289	wt, SGI1^+^, Amp^R^,Chm^R^,Flo^R^,Str^R^,Spt^R^,Sul^R^,Tet^R^,Rif^R^,Kan^S^,Gen^S^,Nal^S^	[22]
ST1321	wt, SGI1^+^, Amp^R^,Chm^R^,Flo^R^,Str^R^,Spt^R^,Sul^R^,Tet^R^,Rif^R^,Kan^S^,Gen^S^,Nal^S^	[22]
ST1323	wt, SGI1^+^, Amp^R^,Chm^R^,Flo^R^,Str^R^,Spt^R^,Sul^R^,Tet^R^,Rif^R^,Kan^S^,Gen^S^,Nal^S^	[22]
ST1367	wt, SGI1^+^, Amp^R^,Chm^R^,Flo^R^,Str^R^,Spt^R^,Sul^R^,Tet^R^,Rif^R^,Kan^S^,Gen^S^,Nal^S^	[22]
ST1369	wt, SGI1^+^, Amp^R^,Chm^R^,Flo^R^,Str^R^,Spt^R^,Sul^R^,Tet^R^,Rif^R^,Kan^S^,Gen^S^,Nal^S^	[22]
ST1373	wt, SGI1^+^, Amp^R^,Chm^R^,Flo^R^,Str^R^,Spt^R^,Sul^R^,Tet^R^,Rif^R^,Kan^S^,Gen^S^,Nal^S^	[22]
ST1375	wt, SGI1^+^, Amp^R^,Chm^R^,Flo^R^,Str^R^,Spt^R^,Sul^R^,Tet^R^,Rif^R^,Kan^S^,Gen^S^,Nal^S^	[22]
ST1388	wt, SGI1^+^, Amp^R^,Chm^R^,Flo^R^,Str^R^,Spt^R^,Sul^R^,Tet^R^,Rif^R^,Kan^S^,Gen^R^,Nal^S^	[22]
ST1579	wt, SGI1^+^, Amp^R^,Chm^R^,Flo^R^,Str^R^,Spt^R^,Sul^R^,Tet^R^,Rif^R^,Kan^S^,Gen^S^,Nal^R^	[22]
ST1712	wt, SGI1^+^, Amp^R^,Chm^R^,Flo^R^,Str^R^,Spt^R^,Sul^R^,Tet^R^,Rif^R^,Kan^S^,Gen^S^,Nal^R^	[22]
ST1713	wt, SGI1^+^, Amp^R^,Chm^R^,Flo^R^,Str^R^,Spt^R^,Sul^R^,Tet^R^,Rif^R^,Kan^S^,Gen^S^,Nal^R^	[22]
ST1714	wt, SGI1^+^, Amp^R^,Chm^R^,Flo^R^,Str^R^,Spt^R^,Sul^R^,Tet^R^,Rif^R^,Kan^S^,Gen^S^,Nal^R^	[22]
ST1772	wt, SGI1^+^, Amp^R^,Chm^R^,Flo^R^,Str^R^,Spt^R^,Sul^R^,Tet^R^,Rif^R^,Kan^S^,Gen^S^,Nal^S^	[22]
ST1773	wt, SGI1^+^, Amp^R^,Chm^R^,Flo^R^,Str^R^,Spt^R^,Sul^R^,Tet^R^,Rif^R^,Kan^S^,Gen^S^,Nal^S^	[22]
ST11S (1,2,3)	SGI1^+^, Amp^S^,Chm^S^,Flo^S^,Str^R^,Spt^R^,Sul^R^,Tet^S^,Rif^R^,Kan^S^,Gen^S^,Nal^S^ derivatives of *S.* T. 1134	This work
ST11A (1,2)	SGI1^+^, Amp^R^,Chm^S^,Flo^S^,Str^S^,Spt^S^,Sul^R^,Tet^S^,Rif^R^,Kan^S^,Gen^S^,Nal^S^ derivatives of *S.* T. 1134	This work
ST14A	SGI1^+^, Amp^R^,Chm^S^,Flo^S^,Str^S^,Spt^S^,Sul^R^,Tet^S^,Rif^R^,Kan^S^,Gen^S^,Nal^S^ derivative of *S.* T. 1288	This work
ST15S	SGI1^+^, Amp^S^,Chm^S^,Flo^S^,Str^R^,Spt^R^,Sul^R^,Tet^S^,Rif^R^,Kan^S^,Gen^S^,Nal^S^ derivative of *S.* T. 1289	This work
ST18S	SGI1^+^, Amp^S^,Chm^S^,Flo^S^,Str^R^,Spt^R^,Sul^R^,Tet^S^,Rif^R^,Kan^S^,Gen^S^,Nal^S^ derivative of *S.* T. 1367	This work
ST19S (1,2)	SGI1^+^, Amp^S^,Chm^S^,Flo^S^,Str^R^,Spt^R^,Sul^R^,Tet^S^,Rif^R^,Kan^S^,Gen^S^,Nal^S^ derivatives of *S.* T. 1369	This work
ST20S	SGI1^+^, Amp^S^,Chm^S^,Flo^S^,Str^R^,Spt^R^,Sul^R^,Tet^S^,Rif^R^,Kan^S^,Gen^S^,Nal^S^ derivative of *S.* T. 1373	This work
ST21S(1,2,3,4,5,6)	SGI1^+^, Amp^S^,Chm^S^,Flo^S^,Str^R^,Spt^R^,Sul^R^,Tet^S^,Rif^R^,Kan^S^,Gen^S^,Nal^S^ derivatives of *S.* T. 1375	This work
ST21A	SGI1^+^, Amp^R^,Chm^S^,Flo^S^,Str^S^,Spt^S^,Sul^R^,Tet^S^,Rif^R^,Kan^S^,Gen^S^,Nal^S^ derivative of *S.* T. 1375	This work
ST28S (1,2)	SGI1^+^, Amp^S^,Chm^S^,Flo^S^,Str^R^,Spt^R^,Sul^R^,Tet^S^,Rif^R^,Kan^S^,Gen^S^,Nal^S^ derivatives of *S.* T. 1773	This work
*S. e.* Enteritidis 11 PT1	wt	[42]

p.c. – personal communication.

Spontaneous excision of SGI1 was tested by nested PCRs specific for the *attB* sequence which is known to re-establish whenever the excision of the island occurred (Fig. 1A). In the first round of PCRs, *attB* was detected as a week 1.0 kb fragment in each SGI1^+^ strain indicating the very low frequency of the SGI1-free *attB* sites in the bacterial population (Fig. 1B). To achieve more intensive signal, the *attB* site was amplified in a second PCR (Fig. 1C). Sequencing of a nested-PCR amplicon (GenBank JQ345501) confirmed the correct excision of SGI1 (Figure S1A).

To test whether SGI1 can be mobilized from the *S.* T. isolates, the R55 helper plasmid was conjugated into the Nal^S^ strains from *Ec*/R55 (Table 1) and the transconjugants were used as donors in crosses with *E. coli* TG90Nal recipient. Transfer of SGI1 into *E. coli* from all 12 *S.* T. strains tested was detectable and its frequency ranged between ca. 0.014–5.9% (Table 2). Total DNA was isolated from several transconjugant colonies from each cross and the samples were analyzed by PCRs (Figure S2A). Two *E. coli* specific PCRs showed that all Str^R^Nal^R^ clones were *E. coli* transconjugants harbouring SGI1. In the majority of transconjugants, SGI1 integrated into the *attB* site located at the 3′ end of *trm*E (*thd*F) gene. In several clones strong *attP* signal was obtained suggesting that SGI1 integrated as tandem repeats as it was detected previously [21]. In two cases (Figure S2B), SGI1 was probably integrated into a secondary attachment site, as both DR specific PCRs were negative and *attB* specific PCR showed that this site is free of SGI1, however, the SGI1 specific PCR for *tet*(G) and the resistance pattern of these clones confirmed that both harbour the island. These results showed that the tested SGI1 copies of Hungarian *S.* T. isolates are all fully competent for excision, integration and mobilization by the helper plasmid R55. Based on the above data, as well as on the resistance pattern of the original and the transconjugant strains and additional PCR and sequencing results (not shown), we supposed that these SGI1 copies are all identical to the prototype of the island [2].

**Table 2:** Transfer frequencies of SGI1 from *S.* T. strains into *E. coli* recipient.

*Donor strain/R55* a	*Donor titer (×10^9^/ml)*	*Recipient titer* b *(×10^9^/ml)*	*Transconjugant titer(×10^7^/ml)*	*Frequency of conjugation (%)*	
				*transconjugant/recipient*	*transconjugant/donor*
ST1134	3.80±0.86	4.05±2.13	19.5±10.7	5.89±2.78	5.17±2.65
ST1233	12.40±4.25	4.10±0.92	8.50±3.60	2.02±0.65	0.73±0.26
ST1288	11.80±4.21	6.10±0.59	16.0±10.5	3.17±2.37	1.92±1.62
ST1289	31.30±4.11	9.33±2.49	0.26±0.12	0.033±0.022	0.008±0.003
ST1321	6.25±3.90	4.05±2.32	16.0±10.5	3.94±0.97	9.50±1.41
ST1323	19.30±6.60	10.70±1.89	0.15±0.10	0.014±0.008	0.007±0.003
ST1367	14.70±4.11	12.70±3.77	0.80±0.28	0.062±0.003	0.054±0.008
ST1369	15.30±7.80	7.57±3.89	0.14±0.09	0.018±0.007	0.010±0.004
ST1373	19.30±9.57	5.73±0.19	3.20±0.75	0.55±0.13	0.22±0.11
ST1375	22.00±2.83	5.87±019	2.13±0.94	0.37±0.17	0.102±0.051
ST1772	12.00±5.89	10.7±1.89	26.7±12.3	2.47±0.90	2.33±0.50
ST1773	11.30±5.73	5.93±0.25	24.0±18.5	4.17±3.38	2.31±1.20
ST21S^c^	8.53±6.64	10.10±7.59	153±65.0	20.00±6.92	27.50±17.5
ST21A/1^c^	9.33±0.94	7.47±0.50	23.3±3.4	3.17±0.69	2.5±0.25
ST28S/1^c^	15.50±4.77	4.33±0.13	25.0±9.9	5.76±2.27	1.78±0.84

a All donor strains harboured the IncA/C helper plasmid R55.

b Recipient strain was the *E. coli* TG90Nal in each cross.

c The three donor strains are representatives of the newly isolated S- and A-type variants.

### Stability of SGI1

Detection of *attB* site in the SGI1^+^ bacterial populations indicated that the island can be excised from the chromosome with a low but detectable frequency. This raises the possibility that SGI1 may be unstable and can segregate. In order to test the stability of the island, all the 17 SGI1^+^ *S.* T. isolates were propagated through ca. 350 generations (43 passages) without selection for SGI1 and the loss of the island was monitored by replica plating. Altogether 12732 and 4021 colonies from the 1^st^ and 43^rd^ passages, respectively, were tested for changes in their antibiotic resistances. Twenty Chm^S^ and Tet^S^ segregants were isolated from 10 strains (Table 3), which all classified into two phenotypes: Amp^R^Str^S^Chm^S^Tet^S^ (A-type) or Str^R^Amp^S^Chm^S^Tet^S^ (S-type). PCRs proved the presence of both the DRL and DRR junctions in these segregants, suggesting that the resistance variations were probably due to deletions inside SGI1 rather than the complete loss of the island. Thus, - contrary to the expectations - we could not isolate any SGI1^−^ clones from the 16753 tested colonies. Additionally, PCR amplification of *attB* from the 1^st^, 11^th^ and 43^rd^ passages did not show detectable accumulation of SGI1^−^ cells in the populations (data not shown). This suggested that SGI1^−^ segregants (if exist) represent a very small proportion of the populations and they have no significant advantage during propagation. On the other hand, the isolated segregants with altered resistance phenotype indicate that the structure of SGI1 is not perfectly conserved and variants emerge with detectable frequency even in a short timescale.

**Table 3:** Phenotypic characterisation of Chm^S^Tet^S^ segregants obtained from long term propagation of SGI1^+^ *S.* T. strains.

*Strain*	*Colonies tested (1^st^/43^rd^ passage)*	*Chm^S^Tet^S^ segregants (1^st^/43^rd^ passage)*	*Remaining resistances* a	*Name*	*attB*	DRL	DRR
ST1134	809/212	3/0	StrSptSull	ST11S/1-3	-	+	+
ST1134	809/212	2/0	AmpSu	ST11A/1-2	-	+	+
ST1233	306/201	0/0	*	*	*	*	*
ST1288	502/234	0/1	AmpSul	ST14A	-	+	+
ST1289	185/132	1/0	StrSptSu	ST15S	-	+	+
ST1321	448/234	0/0	*	*	*	*	*
ST1323	1939/321	0/0	*	*	*	*	*
ST1367	326/185	1/0	StrSptSul	ST18S	-	+	+
ST1369	1742/176	2/0	StrSptSul	ST19S/1-2	-	+	+
ST1373	478/241	1/0	StrSptSul	ST20S	-	+	+
ST1375	1638/183	1/5	StrSptSul	ST21S/1-6	-	+	+
ST1375	1638/183	1/0	AmpSul	ST21A	-	+	+
ST1388	542/241	0/0	*	*	*	*	*
ST1579	385/134	0/0	*	*	*	*	*
ST1712	195/342	0/0	*	*	*	*	*
ST1713	192/296	0/0	*	*	*	*	*
ST1714	178/241	0/0	*	*	*	*	*
ST1772	1220/132	0/0	*	*	*	*	*
ST1773	1647/195	2/0	StrSptSul	ST28S/1-2	-	+	+
Sum total	**12732/4021**	**14/6**					

a Only the SGI1-borne resistances are shown. The AmpSul and StrSptSul phenotypes correspond to the A- and S-type derivatives, respectively.

*no segregants were detected.

### Analysis of the A- and S-type SGI1 variants

First, the mobilization of SGI1 from several A- and S-type segregant clones (ST21S, ST21A/1 and ST28/S1) was tested. The transfer rates proved to be similar to or higher than that of the wt island (Table 2), suggesting that the deletions did not affect the regions necessary for mobilization and transfer functions. Resistance patterns of these variants suggested that deletions eliminating several resistance markers could occur between the directly oriented homologous sequences of the In104 cluster. The 441 bp repeats are located at the 5′ end of ORFs *IntI1* and *groEL/IntI1* bracketing the *aad*A1 (Str^R^/Spt^R^), *floR* (Flo^R^/Chm^R^) and *tet*(G) (Tet^R^) resistance genes, while the 983 bp repeats take place in *qacEΔ1/sulΔ1* and *qacEΔ1/sul1* bracketing the *floR*, *tet*(G) and *bla_PSE-1_* (Amp^R^) resistance genes (Fig. 2A). Primers specific for upstream and downstream sequences of the repeats were applied for verifying the structures of A- and S-type SGI1 segregants (Fig. 2A and Table S1). As expected, PCR primers *f*-*g* amplified a 704 bp fragment from the A-type variants and none from the S-type forms, while primers *d*-*e* did not amplify any fragments from the A-type, but resulted in a 1172 bp fragment from the S-type segregants (Fig. 2B). Sequencing of the PCR products obtained from two of these segregants ST21A/3 and ST21S/1 (GenBank JQ345502 and JQ345503, respectively, both isolated from the wt strain ST1375) confirmed that both deletions occurred between the homologous tracts (Figure S1B and C).

### Entrapment of SGI1 on plasmids

For detailed analysis we decided to integrate SGI1 it into a plasmid. Since SGI1 integration into secondary *attB* sites had been observed, we supposed that the spontaneously excised island could integrate into a conjugative plasmid as well, and the cointegrates then could be mobilized. To test this, the Kan^R^ plasmid pJKI635 (a pRK2013 derivative) was conjugated into 11 SGI1^+^ *S.* T. strains. After a two-day-long incubation at room temperature (to help the accumulation of pJKI635::SGI1 cointegrates), the transconjugants were used as donors in a second cross with an *E. coli* recipient. Although the transfer frequency of pJKI635 ranged between 0.09 to 26.7% of the recipient cells, no pJKI635::SGI1 cointegrates were detected (the frequency was <1.4×10^−6^ among the pJKI635 transconjugants in each cross). A similar negative result was obtained when the *attB* site from *E. coli* chromosome (*attB_Ec_*) was inserted into pJKI635 and large number of colonies harbouring the resulting trap plasmid, pJKI643, was pooled and used as donor in conjugations (the frequency of pJKI643::SGI1 cointegrates was <1.6×10^−7^ per donor in each cross).

After these unsuccessful attempts, the experimental setup was changed. The p15A-based plasmid, pJKI629, containing the *attB* from *S.* T. chromosome (*attB_ST_*) was introduced into *E. coli* TG90Nal and this strain was used as a recipient in crosses with SGI1^+^ donor strains harbouring R55. In this setup, SGI1 mobilized by R55 can integrate into two types of *attB* sites in the recipient: into the single chromosomal copy of *attB_Ec_* and into the 20–30 copies of *attB_ST_* placed on the trap vector pJKI629. In this experiment, the integration site in SGI1^+^ transconjugants could be identified only by PCRs specific for DRR_Ec_ (chromosomal integration) and DRR_ST_ (plasmid integration). After several unsuccessful attempts to detect SGI1 integration in the trap plasmid, we supposed that the severe replication disadvantage of the large and rare cointegrates among the empty trap plasmid copies (46 vs. 3 kb) might have been responsible for the negative results. To decrease this disadvantage, a single copy trap vector pJKI666 was constructed. Applying pJKI666 in a similar experimental setup as described above, we could detect insertions of SGI1 as well as the S- and A-type variants onto the single copy trap plasmid (Table 4). Several transconjugant colonies were assayed by PCR for the integration sites. In almost half of the tested colonies the integration occurred at both available *attB* sites. The chromosomal integration appeared to be more frequent (88–100%), while integration into the trap plasmid ranged between 0–88% (Table 4), but we found two clones (in the case of ST28S/1 donor) where only the plasmid born *attB* site was occupied.

**Table 4:** Capture of SGI1 in pJKI666 trap vector.

*Recipient strain/pJKI666* a	*Donor strain/R55* b	*Donor titer (×10^9^/ml)*	*Recipient titer (×10^9^/ml)*	*Transconjugant titer (×10^8^/ml)*	*Frequency of conjugation (transconjugants/recipients, %)*	*Targeted integration site*	
						*chromosome*	*pJKI666*
TG90Nal	ST1773	20.70±9.57	1.07±0.34	0.13±0.04	1.43±0.69	24/24 (100%)	16/24 (67%)
TG90Nal	ST28S/1	18.50±3.57	1.10±0.64	0.36±0.14	3.96±2.34	21/24 (88%)	21/24 (88%)
TG90Nal	ST21S/1	6.00±2.91	5.95±1.37	2.64±0.66	4.80±2.17	12/12 (100%)	2/12 (17%)
TG90Nal	ST21A	29.5±12.1	4.50±0.57	0.34+0.06	0.78±0.23	12/12 (100%)	0/12 (<8%)
TG1Nal	ST21S/1	17.30±1.89	2.87±0.34	2.42±1.25	8.01±3.72	12/12 (100%)	3/12 (25%)
TG1Nal	ST21A	21.5±6.98	2.50±0.64	0.19±0.04	0.77±0.19	12/12 (100%)	1/12 (8%)

a Recipient strains harboured the single copy trap vector pJKI666.

b All donor strains harboured the IncA/C helper plasmid R55.

### Identification of further SGI1 deletion derivatives

**Figure 3:**
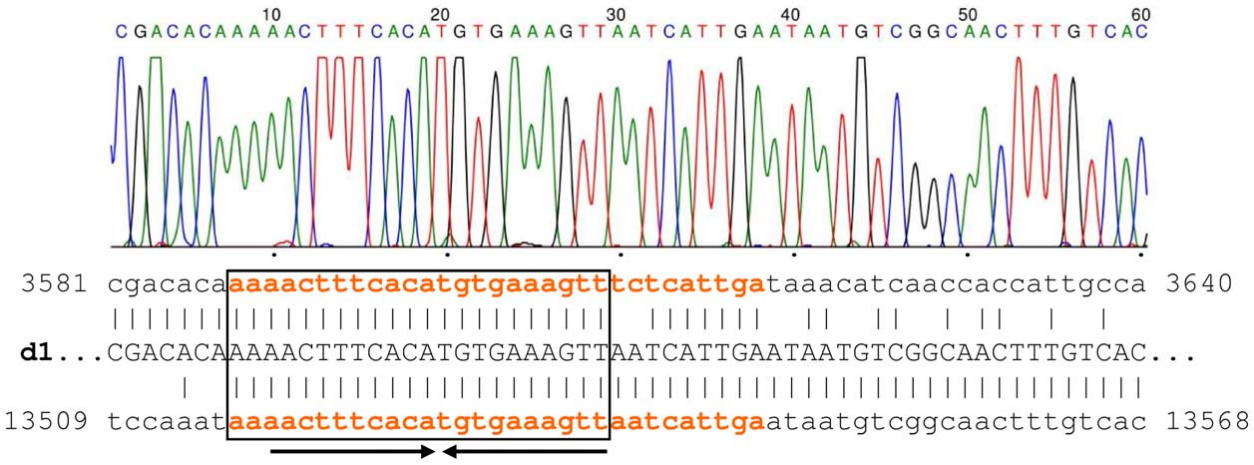
Sequence chart of d1 PCR amplicon obtained from pJKI666::SGI28S/1d1. The alignment of **d1** deletion product and the two homologous regions in 5′ part of SGI1 are shown below the chart, coordinates are indicated according to the SGI1 sequence (GenBank AF261825.2). The 31 bp imperfect direct repeats are highlighted by orange and bold. The 22 bp perfect homology where the crossing over occurred is indicated by frame. The 20 bp palindrome is shown by arrows.

Restriction analysis of the pJKI666::SGI1 cointegrates (not shown) suggested that one of them, derived from ST28S/1, carried a large deletion in the first third of SGI1. We supposed that this new deletion could be the result of recombination between direct repeats, similarly to the S- and A-type deletions. After thorough analysis of the SGI1 sequence, we found 31-bp imperfect direct repeats having a 22 bp perfect palindrome, which are located ca. 10 kb from each other (Fig. 2A and Fig. 3). To test whether the new deletion occurred between these repeats, PCR primers specific to the flanking regions of the repeated sequences were designed. PCR tests for four entrapped SGI1 variants proved that the expected deletion, designated as **d1**, occurred in the S-type SGI1 captured from ST28S/1 (Fig. 2C). Sequencing of the **d1** specific amplicon showed that recombination took place at the first 22 bp perfect homology of the 31 bp repeats (Fig. 3), and removed ca. 10 kb (from 3610 to 13537 bp) including ORFs from S005 to S012. To trace the origin of this deletion, **d1** specific PCR was carried out for the original strain ST28S/1, the donor strain ST28S/1/R55 used in the entrapment cross experiment and several other transconjugants derived from this donor strain. The results showed that ST28S/1 did not contain **d1** in a detectable amount, while its derivative harbouring the R55 helper plasmid was a mixed population carrying the wt and **d1** variant of the S-type SGI1 (data not shown), suggesting that the presence of R55 can enhance the formation of **d1**. The above results indicated that the deleted region did not contain indispensable functions for SGI1 mobilization. This assumption was verified when the mobilization of **d1** and non-**d1** S-type variants (originally derived from ST28S/1d1 and ST21S/1, respectively) were compared, and the presence or absence of **d1** deletion did not significantly influence the transfer frequencies (**d1**: 7.7±8.8×10^−4^ vs. non-**d1**: 12.0±4.6×10^−4^ per recipients).

### Detection of floR deletion

**Figure 4:**
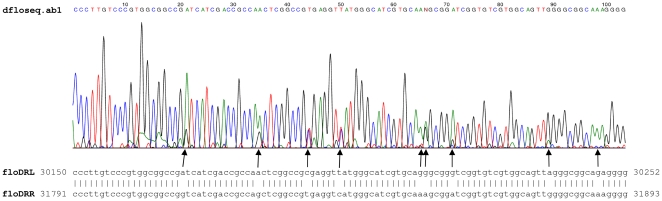
Sequence chart of d*flo* PCR amplicon. The 304 bp **d** ***flo*** PCR fragment obtained from total DNA of strain ST1375 using primers flofor and florev was isolated from agarose gel and directly sequenced with primer flofor. The alignment of floDRL and floDRR are shown below the chart, coordinates are indicated according to the SGI1 sequence (GenBank AF261825.2). The nine mismatched positions and the corresponding signal in the primary sequence are indicated by arrows.

The formation of **d1** deletion by recombination between short direct repeats raised the possibility that a similar process using other direct repeats in SGI1 can lead to additional deletion variants. Since the florfenicol resistance gene *floR* is delimited by 102-bp imperfect repeats (floDRL and floDRR, Fig. 2A), this region appeared to be suitable to test this assumption. PCR primers (flofor-florev) specific for the outer flanking regions of these repeats were designed and the SGI1^+^ ST1375 strain and its derivative harbouring the IncA/C helper plasmid IP40a were tested for the appearance of the variant deleted for *floR* (**d** ***flo***). PCRs resulted in two fragments and sequencing proved that the large fragment (1945 bp) corresponded to the wt situation, while the 304 bp fragment was amplified from **d** ***flo*** templates (Fig. 2D). The appearance of the alternative bases in the sequence of the 304 bp PCR amplicon at the 9 positions, where floDRL and floDRR are mismatched (Fig. 4), suggested that crossing over could have occurred at many positions along the 102-bp homology, thus the resulting subpopulation of **d** ***flo*** variants may represent independent recombination events. Since **d1** derivatives were detected in the presence of R55 conferring resistance for Chm/Flo, Kan, Amp, Gen and Sul, instead, a close relative helper plasmid, IP40a, conferring resistance only for Kan, Amp and Sul was applied to isolate **d** ***flo*** (Amp^R^Str^R^Spt^R^Tet^R^Sul^R^Chm^S^) SGI1 derivatives. IP40a was previously shown to participate in SGI1 mobilization similarly to R55 [20]. Two parallel colonies of strains ST1375 and ST1375/IP40a were grown in LB until the stationary phase under selection for Tet, but without selection for Chm (this prevented the accumulation of A- and S-type derivatives, which are also Chm^S^). The **d** ***flo*** derivatives then were sought by replica plating. Although PCR and sequencing results clearly showed the presence of **d** ***flo*** derivatives in the bacterial populations, no Chm^S^Tet^R^ segregants could be isolated among 9723 and 10420 Tet^R^ colonies of the two strains. This suggested that the frequency of **d** ***flo*** variants in the stationary phase populations was <10^−4^ per Tet^R^ cells.

### Generation of deletion variants of SGI1 and the influence of helper plasmid on this process

**Figure 5:**
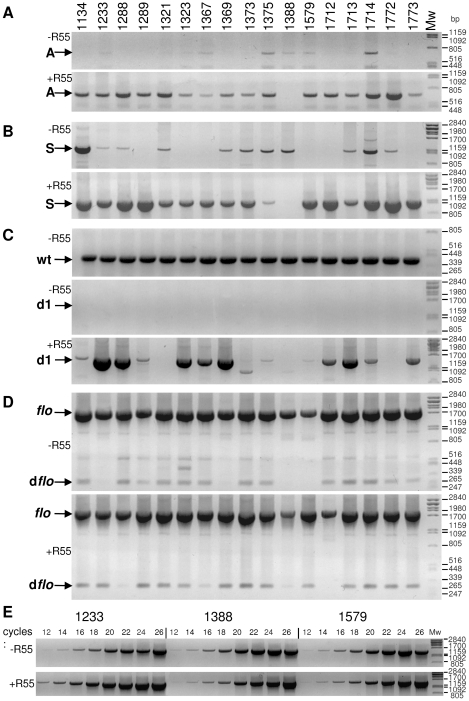
Occurrence of the deletion derivatives of SGI1 in the populations of the 17 original *S.* T. DT104 strains harbouring wt SGI1. PCRs were carried out using total DNA of the original strains and of their derivatives harbouring plasmid R55. Parts **A**, **B**, **C** and **D**: PCR detection of A-, S-, **d1**- and **d** ***flo***-type deletion derivatives, respectively. In part **C**, the first panel shows the wt PCR amplicon. Note that in part **D**, the wt *flo* and **d** ***flo*** amplicons appear together in the same PCRs. Part **E**: Semiquantitative PCR test using 16S rDNA specific primers 16Sfor and 16Srev. PCRs contained templates from ST1233, ST1388 and ST1579 including both the R55- and R55+ series. Cycle numbers applied are shown above the lanes.

Our observation that several S- and A-type deletion variant could be isolated even after the first passages of SGI1^+^ *S.* T. strains suggested that these forms were continuously present as a small fraction in the bacterial populations harbouring wt SGI1. The additional observation that **d1** deletion was only detectable in the presence of R55 hinted at the potential role of the helper plasmid in the generation of deletions. To test these possibilities, extensive PCR analysis were carried out for all the 17 original SGI1^+^ *S.* T. strains and their derivatives harbouring R55. Total DNA was isolated from overnight cultures grown without any selection for SGI1 markers and used in PCRs specific for the four deletions described above (Fig. 5). PCRs were carried out with equal amount of template DNA according to the standard conditions (see Materials and Methods). The results confirmed that the deletion variants were present in most of the populations (except **d1**, which could not be detected in any of the 17 samples) (Fig. 5A–D), although their proportion was obviously very low. The semiquantitative PCR test for the 16S rDNA demonstrated that different intensities (or absence) of PCR amplicons specific for the deletion derivatives were not related to different DNA concentration of the preparations (Fig. 5E). On the other hand, the presence of R55 in the *S*. T. strains caused a striking increase in the intensity of PCR signals for all four deletions in the majority of samples. This effect was most significant in the cases of A-, S- and **d1**-type deletions.

## Discussion

In this study we have investigated the mobility functions and stability of SGI1 in 17 multidrug resistant *S.* T. DT104 strains isolated in Hungary [22]. All the 17 strains showed the common resistance phenotype indicative of SGI1 prototype (ACSSuT) [6], together with additional resistances in several strains (Table 1). The mobility functions (excision, conjugation and integration) of SGI1 copies proved to be intact. The excision was monitored by PCR-detection of *attB* site, which was previously reported to be unsuccessful [6]. The weak *attB*-specific signal obtained in our first PCRs suggests that the occurrence of the SGI1-free *attB* is rather rare indicating that the excision is infrequent event or the reintegration is very effective. The conjugal transfer rate of all the tested strains appeared to be significantly higher (Table 2) compared to the data of a previous report [19], which may be due to the different experimental design and bacterial strains applied. SGI1 predominantly integrated as a single copy into the *attB_Ec_* site at the 3′ end of *E. coli trm*E (*thd*F) gene, while several integrations occurred at secondary sites despite the presence of the intact primary *attB* site and several tandem integrations also occurred as it have been reported earlier [21]. Our results show that the Hungarian *S.* T. DT104 isolates harbour fully competent SGI1 copies in the excision/integration and transfer functions. All the transconjugant *E. coli* clones acquired only the SGI1-specific resistances, which supports that our isolates contain the SGI1 prototype.

SGI1 can spontaneously excise from its chromosomal location and the resulting free circular form appears unable to replicate [19]. The 42.4 kb SGI1 DNA represents ca. 0.86% of the *S.* T. chromosome so its loss might provide some growth advantage for the SGI1^−^ cells. This raises the likelihood that the island segregates in an SGI1^+^ population under non-selective conditions. However, our attempts to isolate SGI1-free segregants of the 17 SGI1^+^ *S.* T. strains and to detect the accumulation of SGI1-free *attB* site by PCR have failed even after 43 passages suggesting that the loss of SGI1 must be very rare and is not significantly beneficial for the cells under the given experimental conditions. The stability of SGI1 has also been reported for two *S. enterica* serovar Paratyphi B dT^+^ isolates, however, the segregation assay was carried out in a smaller scale [23]. The complete loss of pathogenicity islands (PAIs) of the uropathogenic *E. coli* 536 was also investigated [24] and the deletion rate of four of the five PAIs ranged between 10^−5^–10^−6^. In cases where the excision frequency was <10^−5^, the *attP* from the free circular form could not be amplified and the PAI-free *attB* site amplification was not attempted [24]. The facts that after SGI1 excision *attP* and *attB* were also hardly detected by PCR [19 and this work], and that no SGI1^−^ segregants were found by us among more than 16000 colonies suggest that the rate of SGI1-loss falls into a similar range than that of the four PAIs of *E. coli* 536. This stability can be explained by the low activity (or repression) of the site-specific recombination system of SGI1 (*int* and *xis*) responsible for the excision, and/or by the high efficiency of integration reaction leading to nearly 100% reintegration of the excised island. Significantly higher expression level of Int than of Xis [25] suggests that both effects may proceed. Another possibility is that SGI1 provides some advantage for its host. Although SGI1 does not contain genes related to known stability factors such as toxin-antitoxin systems or *kil* functions of several plasmids, one or more of the 44 ORFs, especially those of unknown functions, may contribute to the increased fitness of the SGI1^+^ host, which can compensate for the potential growth advantage of the SGI1^−^ cells.

Although SGI1-free segregants could not be isolated from the 17 *S.* T. strains propagated for ca. 350 generations, two different antibiotic resistance phenotypes were detected. Both arose by deletions between long directly repeated homologous regions in the In104 cluster. Crossing over between the 5′ end of ORFs *IntI1* and *groEL/IntI1* eliminated the *aadA1*, *floR* and *tet*(G) resistance genes led to the formation of A-type variant (retaining only the Amp^R^ and Sul^R^), while the recombination between *qacEΔ1/sulΔ1* and *qacEΔ1/sul1* deleted the *floR*, *tet*(G) and *bla_PSE-1_* resistance genes and resulted in the S-type variant (retaining Str^R^Spt^R^Sul^R^). These variants showed similar transfer activity to that of wt SGI1 (Table 2) and supposedly they are identical to the previously described variants SGI1-B and SGI1-C [3], which were detected in several *Salmonella* serotypes such as Typhimurium DT104, Agona and Paratyphi B and were suggested to arise by a single crossover between the homologous tracts present in the In104 integron region [3], [23], [26,]. Our results demonstrate that the A- and S-type deletion variants are spontaneously arising even in a short timescale in the bacterial populations containing the intact SGI1. The fact that SGI1-B and SGI1-C has been detected in serotypes reported to harbour also the prototype SGI1, further supports this assumption.

The widely accepted reason for spreading the MDR pathogens is the extensive use of antibiotics in human and animal healthcare, however, the observed stability of SGI1 may reflect that pathogens, once acquired such a MDR genomic island, can preserve their resistance determinants for a long time even in the absence of antimicrobial selection pressure. On the other hand, the emergence of SGI1 variants in the bacterial populations may reflect rapid changes occurring even in a single infected patient as well. Although similar changes have not been described so far under natural conditions, it is expected that plasticity of SGI1 could also be proven *in vivo*. In fact, this may not require the presence of artificially introduced helper plasmids, due to the potential helper functions of IncA/C plasmids of the intestinal *E. coli* of animals or man. In general, the conditions responsible for mobilization and variant formation of genomic islands in *Salmonella* have yet to be studied. A recent example shows that the pathogenicity island ROD21 of *Salmonella* Enteritidis can excise from the bacterial chromosome at an increased rate when bacteria reside inside phagocytic cells, suggesting the role of certain *in vivo* environmental conditions such as oxidative stress response against intracellular bacteria [27]. On the other hand, differences in the antimicrobial treatment regimes in different host species may make an essential difference in formation of variants of SGI1, as suggested recently based on Bayesian analyses [28].

For the detailed analysis of SGI1 and its variants, a trap vector system has been developed. After several unsuccessful approaches suggesting that incompatibility and replication handicap of the rare and large plasmid::SGI1 cointegrates may prevent the capture of the island, a single copy trap vector was applied. After conjugal transfer of SGI1 and its A- and S-type variants, integration occurred at both the chromosomal and the single copy plasmid-borne *attB* sites in most cases. Similar approach was applied for SXT integration onto the plasmid pIceCap, although the chromosomal *attB* was deleted from the recipient strain to increase the integration frequency in the plasmid [29]. Restriction and PCR analyses of the entrapped islands showed that their structures corresponded to the published SGI1, SGI1-B or SGI1-C variants, with the exception of a large deletion, named **d1**, affecting the 5′ region of S-type variant derived from ST28S/1 strain. Although **d1** deletion removed the ORFs S005–S012, including ORFs related to plasmid-borne conjugation genes (S005, S011, S012) [2], the conjugative transfer of this variant was not significantly altered. This suggests that these ORFs are not involved in SGI1 transfer and oriT (origin of transfer) locates elsewhere in the island. The 5′ region was also affected in an SGI1 variant described from *S. e.* serovar Kentucky, where the insertion of IS*Vch*4 element promoted deletion from S005 to S009, however the transfer potential of this variant has not been tested [13].

We report a further rare deletion (**d** ***flo***) occurring between the 102 bp imperfect direct repeats bracketing *floR* gene. Importantly, A-, S- and **d** ***flo***-type deletions were easily detectable by PCR in the bacterial populations while **d1** was identified only in the presence of the helper plasmid R55. As the introduction of R55 caused significant increase in the formation of all variants, we suppose that the helper plasmid or the conjugal transfer process itself could promote deletion formation. In the lack of R55, deletions resulting in A- and S-type variants probably occur via RecA-dependent homologous recombination between the long direct repeats. In contrast, **d1** and **d** ***flo*** would require recombinations between direct repeats where the perfect homologies are shorter than 25 bp. This suggests that RecA-independent recombination pathways are involved [30], [31]. Short-homology dependent illegitimate recombination was reported to occur during plasmid replication [32], [33], however, SGI1 is unlikely to replicate itself, therefore deletions can form in a different way, e.g. via chromosomal replication.

One of the possible explanations for the elevated recombination frequency observed in the presence of helper plasmid is that the conjugation helper initiates rolling circle replication of the excised SGI1, which presumably favours the copy-choice recombination between direct repeats [34]. Alternatively, the role of the λ Red-like recombinase RecT (Bet) expressed by the IncA/C helper plasmids (e.g. orf_0123 in plasmid pIP1202, GenBank CP000603.1) can not be excluded either. Since the recombination events are presumably rare, the significant increase of the deletion variants in the presence of the helper plasmid may hint at a growth advantage provided by these variants to their host, which can explain their relative accumulation. In any case, our results indicate that recombination mechanisms of the host cell continuously generate SGI1 deletion variants. The increased level of recombination in the presence of the conjugative helper plasmid emphasizes furthermore that conjugal transfer of SGI1 assisted by IncA/C plasmids is not only important in its spreading, but significantly contributes to the evolution of the island by promoting the appearance of new variants.

In summary, we demonstrated that SGI1 is able to excise spontaneously from the chromosome in all of the investigated Hungarian SGI1^+^ *S.* T. strains, and we could detect a high frequency of SGI1 transfer and integration into the *E. coli* chromosome. Additionally, a trap vector has been constructed and successfully applied to capture the island on a plasmid via its conjugal transfer. Monitoring of segregation of SGI1 indicated high stability of the island. SGI1-free segregants did not accumulate during long-term propagation, but several SGI1 variants could be obtained. Most of them appeared to be identical to SGI1-B and SGI1-C, but two new variants have also been detected: one with a ca. 10 kb deletion between the short direct repeats in the SGI1 backbone (**d1**), and a further deletion variant lacking the *flo*R gene (**d** ***flo***). We have also noticed that the presence of the conjugation IncA/C helper plasmid increased the formation of these deletion variants considerably, indicating that such helper plasmids are not only involved in the horizontal spreading but also in the evolution of SGI1.

## Materials and Methods

### Bacterial strains and microbial techniques

Bacteria (Table 1) were grown at 37°C in LB and stock cultures were stored at −70°C in LB supplemented with 20% glycerol. The final concentration of antibiotics used were: ampicillin (Amp) 150 µg/ml, chloramphenicol (Chm) 20 µg/ml, gentamicin (Gen) 25 µg/ml, kanamycin (Kan) 30 µg/ml, nalidixic acid (Nal) 20 µg/ml, rifampicin (Rif) 20 µg/ml, spectinomycin (Spt) 50 µg/ml, streptomycin (Str) 50 µg/ml, tetracycline (Tet) 10 µg/ml. Prototrophy was tested on M9 plates supplemented with 0.4% glucose. Nal^R^ derivatives of TG1 [35] and TG90 [36] were selected on LB+Nal plates.

Conjugation was carried out as follows: 100 µl of stationary phase LB cultures of donor and recipient cells supplemented with the appropriate antibiotics were mixed, centrifuged, washed with 0.5 ml 0.9% NaCl solution, spread on LB agar plates and incubated overnight (ON) at 37°C. The bacterial lawn was suspended in 4 ml 0.9% NaCl solution, 1–10^7^× dilutions were prepared in 96-well plates and 5 µl of dilutions were dropped onto selective LB plates to determine the titers of donor, recipient and transconjugant cells. Conjugation frequency was calculated from data of 3–6 parallel crosses.

For comparison of mobilization of **d1**- and non-**d1** S-type SGI1, both islands were chromosomally integrated into TG1Nal strain by crosses using the donor strains ST28S/1/R55 and ST21S/1/R55, respectively. Then, R55 was conjugated into the TG1Nal::SG1 transconjugants and these strains were applied as donors in crosses with TG2 recipient.

### Segregation test for SGI1

Three parallel cultures were founded from single colonies in 3 ml LB medium for all the 17 SGI1^+^ *S.* T. strains and grown at 37°C under vigorous shaking without selection for the SGI1 resistance markers. Ten µl of the stationary cultures was serially transferred into 3 ml fresh LB medium (2 passages per day, 300× dilution per passage), thus one passage represented ca. 8 generations. Cultures from the 1^st^ and 43^rd^ passages were plated on LB agar and replica-plated onto LB+Tet and LB+Chm plates. Chm^S^ and Tet^S^ derivatives were tested for the SGI1 resistance markers (except Sul^R^) and the presence/absence of DRL, DRR and *attB* was monitored by PCR.

### DNA techniques and PCR

In general, standard DNA procedures [37] were applied. All cloning were carried out in *E. coli* strain TG1. Total DNA was purified as described earlier [38]. Enzymes were purchased from Fermentas, New England Biolabs and Amersham, chemicals from Sigma, Roth and Roche. Sequencing was performed on ABI Prism 3100 Genetic Analyzer (Perkin Elmer). For the sequence analyses GCG software package was applied [39].

Standard PCRs were carried out in 25 µl containing 2.5 µl 10× buffer and 1U Taq polymerase (NEB), 10 µM of primers (Table S1), 0.2 mM dNTP, 2 mM MgCl_2_ and 1 µl of 10× diluted total DNA or 100× diluted plasmid DNA or 2.5 µl stationary phase LB culture (for colony PCRs). Standard cycling conditions were 94°C for 2 min followed by 35 cycles of 94°C for 20 s, 55°C for 30 s, 72°C for 2 min, followed by a final extension at 72°C for 5 min (alterations are indicated in the text).

### Detection of SGI1 excision by nested PCR specific for the attB site

First PCR was carried out in 25 µl containing 2.5 µl 10× Thermopolymerase buffer and 1U Taq polymerase (NEB), 50 µM of primers attsgi1for and C9-L1, 0.2 mM dNTP, 2.5 mM MgCl_2_ and 1 µl of 10× diluted total DNA. In the second PCR, 1 µl sample from the first PCR was amplified with primers U7-L12 and C9-L2 under the same reaction conditions. Cycling for both PCRs was 94°C for 2 min followed by 35 cycles of 94°C for 20 s, 55°C for 30 s, 72°C for 1 min, followed by a final extension at 72°C for 5 min.

### Cloning of attB sites from S. Typhimurium and E. coli and the construction of trap vectors for SGI1 entrapment

The *attB* site was amplified from total DNA of *E. coli* TG1 with primers attsgi1for and attsgi1rev (*attB_Ec_*–391 bp) and of *S.* T. LT2 MA1703 using primers attsgi1for and C9-L1 (*attB_ST_*–1031 bp). The *attB* fragments were cloned with longer flanking regions of the 18 bp integration site to provide all the potential sequences (if any) that are important for integration of SGI1. Both PCR fragments were inserted into the *Xba*I site of pJKI88 [38] resulting in pJKI627 for *attB_Ec_* and pJKI629 for *attB_ST_*, then both clones were sequenced. *attB_Ec_* from pJKI627 was joined to the Tet^R^ gene from pBR322 (pJKI639) and the Tet^R^-*attB_Ec_* cassette from pJKI639 was inserted into the unique *Xba*I site of a pRK2013 [40] derivative plasmid pJKI635 (J. Kiss, unpublished), resulting in pJKI643. *attB_ST_* from pJKI629 was joined to the Gen^R^ gene from pJQ200SK [41] through several cloning steps and the *attB_ST_*-Gen^R^ cassette was inserted into pBeloBac11 (NEB) with *Eco*RI resulting in the Gen^R^Chm^S^ trap vector pJKI666 (see also Table 5, more detailed plasmid descriptions are available upon request).

**Table 5:** Plasmids used in the experiments.

*Plasmids*	*Resistance*	*Replication*	*Derivative of*	*Other relevant features*	
pJKI627	Kan^R^	p15A	pACYC177	*attB_Ec_*	This work
pJKI629	Kan^R^	p15A	pACYC177	*attB_St_*	This work
pJKI635	Kan^R^	colE1	pRK2013	tra+	This work
pJKI643	Kan^R^,,Tet^R^	colE1	pRK2013	tra+, *attB_Ec_*	This work
pJKI666	Gen^R^	F	pBeloBac11	*attB_St_*	This work
R55	Amp^R^Kan^R^Chl^R^Flo^R^Gen^R^Sul^R^	IncA/C	-	tra+	[20]
IP40a	Amp^R^Kan^R^Sul^R^	IncA/C	-	tra+	[20]

### Capture of SGI1 in plasmids

The Kan^R^ conjugative plasmid pJKI635 (a pRK2013 derivative) was introduced into ST1134, ST1233, ST1288, ST1289, ST1323, ST1367, ST1369, ST1373, ST1375, ST1388, ST1772 and ST1773. Single Chm^R^Amp^R^Kan^R^ colonies from all conjugations kept for two days at room temperature were grown in LB+Chm+Amp+Kan ON at 37°C and used as donors in a second conjugation with TG90Nal recipient. Transconjugants for pJKI635 and pJKI635::SGI1 cointegrates were selected on LB+Nal+Kan and LB+Nal+Chm, respectively. Plasmid pJKI643, a derivative of pJKI635 containing the *attB_Ec_* was introduced into ST1289, ST1323, ST1367 and ST1772. 4–500 Kan^R^Chm^R^ transconjugant colonies of the four *S.* T. strains harbouring pJKI643 were incubated at room temperature for 4 days, then pooled in 4 ml LB+Kan+Chm, grown at 37°C for 2 hours and the four pooled cultures were used as donors in a second conjugation with TG90Nal recipient. Transconjugants for pJKI643::SGI1 cointegrates were selected on LB+Nal+Kan+Chm.

The IncA/C plasmid R55, conferring Kan^R^Gen^R^Chm^R^Flo^R^Amp^R^, was introduced into the SGI1^+^ strains ST1134, ST1233, ST1288, ST1289, ST1323, ST1367, ST1369, ST1373, ST1375, ST1388, ST1772, ST1773, ST28S/1, ST21S/1 and ST21A by conjugation from an *E. coli* strain harbouring R55 (A. Cloeckaert, pers. comm.). Str^R^Spt^R^Kan^R^Gen^R^ transconjugant colonies were streaked on selective LB plates (except ST21A/R55, which was selected on M9+glucose+Kan+Chm plate) and single colonies showing all SGI1 and R55 specific resistance markers were used as donors in crosses with TG90Nal recipient harbouring the trap vector pJKI629 or pJKI666 containing the *attB_Ec_* or *attB_ST_*, respectively.

Four *S.* T. strains (ST1773, ST28S/1, ST21S/1 and ST21A) harbouring the R55 helper plasmid were used as donors in crosses with TG90Nal/pJKI666 or TG1Nal/pJKI666 recipients. The SGI1 transconjugants were selected on LB+Nal+Str plates (Except A-type SGI1 transconjugants from ST21A/R55 donors, which were selected on LB+Nal + 300 µg/ml Amp that is selective against R55, then the Nal^R^Amp^R^Chm^S^Kan^S^ transconjugants were selected to exclude all R55 transconjugants). The TG90Nal::SGI1 transconjugant colonies were tested by PCR for the integration site using primer pairs RJ2 and C9-L2 (characteristic for DRR_ST_, the integration into the *attB_ST_* on the trap vector), and RJ2 and attsgi1rev (characteristic for DRR*_Ec_*, the integration into the chromosomal *attB_Ec_*).

## Supporting Information

Figure S1 **Sequence and alignment of PCR amplicons.** **A.** Sequence of the nested PCR fragment representing the *att*B sequence obtained from the SGI1^+^ strain ST1289. The PCR was carried out using primers U7-L12 and C9-L2 (for primers see Table S1) and sequenced with U7-L12. The alignment shows that the PCR product corresponds to the 3′-end of *trm*E (*thd*F) gene and its downstream flanking region of the SGI1-free *S.* T. LT2 chromosome. The *att*B site is highlighted by orange in bold. **B.** Sequence of the PCR fragment obtained from the A-type variant clone ST21A/3 using primers sgi1Adelfor and sgi1Adelrev. The alignment shows that the 704 bp PCR product spans the 441 bp homology of the 5′ part of *IntI1* and *groEL/IntI1* (highlighted by green and bold) from the upstream flanking region of the first to the downstream flanking region of the second repeat (the alignment to the second repeat is shown partially), which proves that A-type deletion occurred between the directly repeated perfect homologies. **C.** Sequence of the PCR fragment obtained from the S-type variant clone ST21S/1 using primers sgi1Sdelfor and sgi1Sdelrev. The alignment shows that the 1172 bp PCR product spans the 983 bp homology in *qacEΔ1/sulΔ1* and *qacEΔ1/sul1* (highlighted by blue and bold) from the upstream flanking region of the first to the downstream flanking region of the second repeat (the alignment to the second repeat is shown partially), which proves that S-type deletion occurred between these directly repeated perfect homologies.
(PDF)

Figure S2 **PCR analysis of the SGI1 transconjugant** ***E. coli*** **TG90Nal strains.** **A.** Lanes 1–12 show the PCR amplicons from the total DNA of 12 randomly chosen Nal^R^Str^R^ TG90Nal transconjugant colonies, where the donor strains (all harbouring R55) were ST1134, ST1233, ST1288, ST1289, ST1321, ST1323, ST1367, ST1369, ST1373, ST1375, ST1772 or ST1773, respectively. Lane 13: TG90Nal recipient, Lane 14: ST1773/R55 donor. Primers were used as follows: DRL – attsgi1for-LJ2, DRREc – RJ2-attsgi1rev, *attBEc* – attsgi1for-attsgi1rev, *attP* – LJ2-RJ2, *tet*(G) – tetGfor-tetGrev, IS*30*C – IS30Cfor-IS30Crev. Positive signals for the DRL, DRREc, and *tet*(G) prove the presence of SGI1, while bands for DRREc and IS*30*C (specific for the chromosomal copy of *E. coli* IS element IS*30*, IS*30*C [1]) show that the sample colonies were *E. coli*. The faint positive signal for *attB_Ec_* probably came from the *attB* site left behind SGI1 by its spontaneous excision as observed with the original *Salmonella* strains (see Fig. 1b). The expected fragment sizes are indicated. **B.** PCR tests for two representative Nal^R^Str^R^ TG90Nal transconjugant colonies, where the insertion occurred outside of the primary *attB* site. Lanes 1–2: two transconjugant colonies, where the donor strains were ST1289 and ST1773, respectively. Lanes 3–4 are TG90Nal recipient and ST1773/R55 donor, respectively. Reference: 1. Umeda M, Ohtsubo E (1990) Mapping of insertion element IS*30* in the *Escherichia coli* K12 chromosome. Mol Gen Genet 222: 317–322.
(PDF)

Table S1 **Oligonucleotide primers used for PCR.**
(PDF)
