# Supplementary material for: Stability, Entrapment and Variant Formation of Salmonella Genomic Island 1
Source: PLoS One. 2012 Feb 23;7(2):e32497. doi: 10.1371/journal.pone.0032497 (PMC3285670; doi:10.1371/journal.pone.0032497)
Supplement: Figure S1 — Sequence and alignment of PCR amplicons. A. Sequence of the nested PCR fragment representing the attB sequence obtained from the SGI1+ strain ST1289. The PCR was carried out using primers U7-L12 and C9-L2 (for primers see Table S1) and sequenced with U7-L12. The alignment shows that the PCR product corresponds to the 3′-end of trmE (thdF) gene and its downstream flanking region of the SGI1-free S. T. LT2 chromosome. The attB site is highlighted by orange in bold. B. Sequence of the PCR fragment obtained from the A-type variant clone ST21A/3 using primers sgi1Adelfor and sgi1Adelrev. The alignment shows that the 704 bp PCR product spans the 441 bp homology of the 5′ part of IntI1 and groEL/IntI1 (highlighted by green and bold) from the upstream flanking region of the first to the downstream flanking region of the second repeat (the alignment to the second repeat is shown partially), which proves that A-type deletion occurred between the directly repeated perfect homologies. C. Sequence of the PCR fragment obtained from the S-type variant clone ST21S/1 using primers sgi1Sdelfor and sgi1Sdelrev. The alignment shows that the 1172 bp PCR product spans the 983 bp homology in qacEΔ1/sulΔ1 and qacEΔ1/sul1 (highlighted by blue and bold) from the upstream flanking region of the first to the downstream flanking region of the second repeat (the alignment to the second repeat is shown partially), which proves that S-type deletion occurred between these directly repeated perfect homologies. (PDF) [file pone.0032497.s001.pdf]

A. Sequence of one of the nested PCR fragments representing the *att*B site obtained from strain ST1289 (GenBank JQ345501)

```
  1   GGCGCATGGG CGGGGGAACT GCTGGCGGAA GAGTTGCGCC TGGCGCAGCA
 51   AAGCTTAAGC GAGATTACCG GCGAGTTTAC CTCCGACGAC CTGCTGGGAC
101   GGATTTTCTC CAGCTTCTGT ATCGGTAAGT AAGTTTTAGT CCACCCTCGT
151   CAGTGAACGT TCACTAACCC GCATTAACTG CCTGTTAACG CGGGTTTTTA
201   CTTTCCATAC TCGTCCATAG TCGTGCGGGT TCATCCGCGA TTTTTGTGTC
251   CTGAATTGTC CCCATCTTTA AATCCCTCGT GTACCATTGT TTACTTTTTG
301   ATCTAAAAAT TACATCAAAT GAACACAAAA AACGGTATAA AAAACGCTCA
351   AATATCCCTT CTCATGGACA CACTATAGAG GTGAGATATG GCACTTTCAG
401   ATACCAAATT ACGCNCACTT
```

Sequence alignment of the nested PCR fragment to the *thd*F gene and its downstream region of *S. e.* Typhimurium LT2 whole genome sequence (GenBank AE006468.1).

```
                                .         .         .         .         .         .
attB       1    GGCGCATGGGCGGGGGAACTGCTGGCGGAAGAGTTGCGCCTGGCGCAGCAAAGCTTAAGC 60
                ||||||||||||||||||||||||||||||||||||||||||||||||||||||||||||
LT2 4049446    ggcgcatgggcgggggaactgctggcggaagagttgcgcctggcgcagcaaagcttaagc
4049505

                                .         .         .         .         .         .
attB      61    GAGATTACCGGCGAGTTTACCTCCGACGACCTGCTGGGACGGATTTTCTCCAGCTTCTGT 120
                ||||||||||||||||||||||||||||||||||||||||||||||||||||||||||||
LT2 4049506    gagattaccggcgagtttacctccgacgacctgctgggacggattttctccagcttctgt
4049565

                                .         .         .         .         .         .
attB     121    ATCGGTAAGTAAGTTTTAGTCCACCCTCGTCAGTGAACGTTCACTAACCCGCATTAACTG 180
                ||||||||||||||||||||||||||||||||||||||||||||||||||||||||||||
LT2 4049566    atcggtaagtaagttttagtccaccctcgtcagtgaacgttcactaacccgcattaactg
4049625

                                .         .         .         .         .         .
attB     181    CCTGTTAACGCGGGTTTTTACTTTCCATACTCGTCCATAGTCGTGCGGGTTCATCCGCGA 240
                ||||||||||||||||||||||||||||||||||||||||||||||||||||||||||||
LT2 4049626    cctgttaacgcgggtttttactttccatactcgtccatagtcgtgcgggttcatccgcga
4049685

                                .         .         .         .         .         .
attB     241    TTTTTGTGTCCTGAATTGTCCCCATCTTTAAATCCCTCGTGTACCATTGTTTACTTTTTG 300
                ||||||||||||||||||||||||||||||||||||||||||||||||||||||||||||
LT2 4049686    tttttgtgtcctgaattgtccccatctttaaatccctcgtgtaccattgtttactttttg
4049745

                                .         .         .         .         .         .
attB     301    ATCTAAAAATTACATCAAATGAACACAAAAAACGGTATAAAAAACGCTCAAATATCCCTT 360
                ||||||||||||||||||||||||||||||||||||||||||||||||||||||||||||
LT2 4049746    atctaaaaattacatcaaatgaacacaaaaaacggtataaaaaacgctcaaatatccctt
4049805

                                .         .         .         .         .         .
attB     361    CTCATGGACACACTATAGAGGTGAGATATGGCACTTTCAGATACCAAATTACGCNCACTT 420
                |||||||||||||||||||||||||||||||||||||||||||||||||||||| |||||
LT2 4049806    ctcatggacacactatagaggtgagatatggcactttcagataccaaattacgcacactt
4049865
```

B. Sequence of the PCR fragment obtained from the A-type variant clone ST21A/3 (GenBank JQ345502).

```
  1   CCGAGGATGC GAACCACTTC ATCCGGGGTC AGCACCACCG GCAAGCGCCG
 51   CGACGGCCGA GGTCTTCCGA TCTCCTGAAG CCAGGGCAGA TCCGTGCACA
101   GCACCTTGCC GTAGAAGAAC AGCAAGGCCG CCAATGCCTG ACGATGCGTG
151   GAGACCGAAA CCTTGCGCTC GTTCGCCAGC CAGGACAGAA ATGCCTCGAC
201   TTCGCTGCTG CCCAAGGTTG CCGGGTGACG CACACCGTGG AAACGGATGA
251   AGGCACGAAC CCAGTGGACA TAAGCCTGTT CGGTTGGTAA GCTGTAATGC
301   AAGTAGCGTA TGCGCTCACG CAACTGGTCC AGAACCTTGA CCGAACGCAC
351   CGGTGGTAAC GGCGCACTGG CGGTTTTCAT GGCTTGTTAT GACTGTTTTT
```

```
401  TTGTACAGTC TATGCCTCGG GCATCCAAGC AGCAAGCGCG TTACGCCGTG
451  GGTCGATGTT TGATGTTATG GAGCAGCAAC GATGTTACGC AGCAGGGCAG
501  TCGCCCTAAA ACAAAGTTAG CCATATTATG GAGCCTCATG CTTTTATATA
551  AAATGTGTGA CAATCAAAAT TATGGGGTTA CTTACATGAA GTTTTTATTG
601  GCATTTTCGC TTTTAATACC ATCCGTGGTT TTTGCAAGTA GTTCAAAGTT
651  T
```

Sequence alignment of the PCR fragment obtained from the A-type variant clone ST21A/3 to the SGI1 sequence (GenBank AF261825.2).

```
                      .         .         .         .         .
ST21A      1 .......................CCGAGGATGCGAACCACTTCATCCGGG 27
                                    |||||||||||||||||||||||||||
SGI1   27450 aacgatgctcgccttccagaaaaccgaggatgcgaaccacttcatccggg 27499
                      .         .         .         .         .
ST21A     28 GTCAGCACCACCGGCAAGCGCCGCGACGGCCGAGGTCTTCCGATCTCCTG 77
             ||||||||||||||||||||||||||||||||||||||||||||||||||
SGI1   27500 gtcagcaccaccggcaagcgccgcgacggccgaggtcttccgatctcctg 27549
                      .         .         .         .         .
ST21A     78 AAGCCAGGGCAGATCCGTGCACAGCACCTTGCCGTAGAAGAACAGCAAGG 127
             ||||||||||||||||||||||||||||||||||||||||||||||||||
SGI1   27550 aagccagggcagatccgtgcacagcaccttgccgtagaagaacagcaagg 27599
                      .         .         .         .         .
ST21A    128 CCGCCAATGCCTGACGATGCGTGGAGACCGAAACCTTGCGCTCGTTCGCC 177
             ||||||||||||||||||||||||||||||||||||||||||||||||||
SGI1   27600 ccgccaatgcctgacgatgcgtggagaccgaaaccttgcgctcgttcgcc 27649
                      .         .         .         .         .
ST21A    178 AGCCAGGACAGAAATGCCTCGACTTCGCTGCTGCCCAAGGTTGCCGGGTG 227
             ||||||||||||||||||||||||||||||||||||||||||||||||||
SGI1   27650 agccaggacagaaatgcctcgacttcgctgctgcccaaggttgccgggtg 27699
                      .         .         .         .         .
ST21A    228 ACGCACACCGTGGAAACGGATGAAGGCACGAACCCAGTGGACATAAGCCT 277
             ||||||||||||||||||||||||||||||||||||||||||||||||||
SGI1   27700 acgcacaccgtggaaacggatgaaggcacgaacccagtggacataagcct 27749
                      .         .         .         .         .
ST21A    278 GTTCGGTTGGTAAGCTGTAATGCAAGTAGCGTATGCGCTCACGCAACTGG 327
             ||||||||||||||||||||||||||||||||||||||||||||||||||
SGI1   27750 gttcggttggtaagctgtaatgcaagtagcgtatgcgctcacgcaactgg 27799
                      .         .         .         .         .
ST21A    328 TCCAGAACCTTGACCGAACGCACCGGTGGTAACGGCGCACTGGCGGTTTT 377
             |||||||||||||||||||||| |||||||||||||||| ||||||||||
SGI1   27800 tccagaaccttgaccgaacgcagcggtggtaacggcgcagtggcggtttt 27849
                      .         .         .         .         .
ST21A    378 CATGGCTTGTTATGACTGTTTTTTTGTACAGTCTATGCCTCGGGCATCCA 427
             ||||||||||||||||||||||||||||||||||||||||||||||||||
SGI1   27850 catggcttgttatgactgtttttttgtacagtctatgcctcgggcatcca 27899
                      .         .         .         .         .
ST21A    428 AGCAGCAAGCGCGTTACGCCGTGGGTCGATGTTTGATGTTATGGAGCAGC 477
             ||||||||||||||||||||||||||||||||||||||||||||||||||
SGI1   27900 agcagcaagcgcgttacgccgtgggtcgatgtttgatgttatggagcagc 27949
                      .         .         .         .         .
ST21A    478 AACGATGTTACGCAGCAGGGCAGTCGCCCTAAAACAAAGTTAGCCATATT 527
             ||||||||||||||||||||||||||||||||||||||||||| |||  |
SGI1   27950 aacgatgttacgcagcagggcagtcgccctaaaacaaagttagacatcat 27999
                      .         .         .         .         .
ST21A    528 ATGGAGCCTCATGCTTTTATATAAAATGTGTGACAATCAAAATTATGGGG 577
                |||  |   | |        |||| |   || | | |    | | |
SGI1   28000 ...gagggtagcggtgaccatcgaaatttcgaaccaactatcagaggtgc 28046

...
...
```

```
                 .         .         .         .         .
ST21A   456 ATGTTTGATGTTATGGAGCAGCAACGATGTTACGCAGCAGGGCAGTCGCC 505
            ||||||||||||||||||||||||||||||||||||||||||||||||||
SGI1  37200 atgtttgatgttatggagcagcaacgatgttacgcagcagggcagtcgcc 37249
                 .         .         .         .         .
ST21A   506 CTAAAACAAAGTTAGCCATATTATGGAGCCTCATGCTTTTATATAAAATG 555
            ||||||||||||||||||||||||||||||||||||||||||||||||||
SGI1  37250 ctaaaacaaagttagccatattatggagcctcatgcttttatataaaatg 37299
                 .         .         .         .         .
ST21A   556 TGTGACAATCAAAATTATGGGGTTACTTACATGAAGTTTTTATTGGCATT 605
            ||||||||||||||||||||||||||||||||||||||||||||||||||
SGI1  37300 tgtgacaatcaaaattatggggttacttacatgaagtttttattggcatt 37349
                 .         .         .         .         .
ST21A   606 TTCGCTTTTAATACCATCCGTGGTTTTTGCAAGTAGTTCAAAGTTT.... 651
            ||||||||||||||||||||||||||||||||||||||||||||||
SGI1  37350 ttcgcttttaataccatccgtggtttttgcaagtagttcaaagtttcagc 37399
```

C. Sequence of the PCR fragment obtained from the S-type variant clone ST21S/1 (GenBank JQ345503).

```
   1  TTCGTTCAAG CCGACCGCGC TACGCGCGGC GGCTTAACTC CGGCGTTAGA
  51  TGCACTAAGC ACATAATTGC TCACAGCCAA ACTATCAGGT CAAGTCTGCT
 101  TTTATTATTT TTAAGCGTGC ATAATAAGCC CTACACAAAT TGGGAGATAT
 151  ATCATGAAAG GCTGGCTTTT TCTTGTTATC GCAATAGTTG GCGAAGTAAT
 201  CGCAACATCC GCATTAAAAT CTAGCGAGGG CTTTACTAAG CTTGCCCCTT
 251  CCGCCGTTGT CATAATCGGT TATGGCATCG CATTTTATTT TCTTTCTCTG
 301  GTTCTGAAAT CCATCCCTGT CGGTGTTGCT TATGCAGTCT GGTCGGGACT
 351  CGGCGTCGTC ATAATTACAG CCATTGCCTG GTTGCTTCAT GGGCAAAAGC
 401  TTGATGCGTG GGGCTTTGTA GGTATGGGGC TCATAATTGC TGCCTTTTTG
 451  CTCGCCCGAT CCCCATCGTG GAAGTCGCTG CGGAGGCCGA CGCCATGGTG
 501  ACGGTGTTCG GCATTCTGAA TCTCACCGAG GACTCCTTCT TCGATGAGAG
 551  CCGGCGGCTA GACCCCGCCG GCGCTGTCAC CGCGGCGATC GAAATGCTGC
 601  GAGTCGGATC AGACGTCGTG GATGTCGGAC CGGCCGCCAG CCATCCGGAC
 651  GCGAGGCCTG TATCGCCGGC CGATGAGATC AGACGTATTG CGCCGCTCTT
 701  AGACGCCCTG TCCGATCAGA TGCACCGTGT TTCAATCGAC AGCTTCCAAC
 751  CGGAAACCCA GCGCTATGCG CTCAAGCGCG GCGTGGGCTA CCTGAACGAT
 801  ATCCAAGGAT TTCCTGACCC TGCGCTCTAT CCCGATATTG CTGAGGCGGA
 851  CTGCAGGCTG GTGGTTATGC ACTCAGCGCA GCGGGATGGC ATCGCCACCC
 901  GCACCGGTCA CCTTCGACCC GAAGACGCGC TCGACGAGAT TGTGCGGTTC
 951  TTCGAGGCGC GGGTTTCCGC CTTGCGACGG AGCGGGGTCG CTGCCGACCG
1001  GCTCATCCTC GATCCGGGGA TGGGATTTTT CTTGAGCCCC GCACCGGAAA
1051  CATCGCTGCA CGTGCTGTCG AACCTTCAAA AGCTGAAGTC GGCGTTGGGG
1101  CTTCCGCTA
```

Sequence alignment of the PCR fragment obtained from the S-type variant clone ST21S/1 to the SGI1 sequence (GenBank AF261825.2).

```
                 .         .         .         .         .
ST21S     1 TTCGTTCAAGCCGACCGCGCTACGCGCGGCGGCTTAACTCCGGCGTTAGA 50
            ||||||||||||||||||||||||||||||||||||||||||||||||||
SGI1  28800 ttcgttcaagccgaccgcgctacgcgcggcggcttaactccggcgttaga 28849
                 .         .         .         .         .
ST21S    51 TGCACTAAGCACATAATTGCTCACAGCCAAACTATCAGGTCAAGTCTGCT 100
            ||||||||||||||||||||||||||||||||||||||||||||||||||
SGI1  28850 tgcactaagcacataattgctcacagccaaactatcaggtcaagtctgct 28899
                 .         .         .         .         .
ST21S   101 TTTATTATTTTTAAGCGTGCATAATAAGCCCTACACAAATTGGGAGATAT 150
            ||||||||||||||||||||||||||||||||||||||||||||||||||
SGI1  28900 tttattatttttaagcgtgcataataagccctacacaaattgggagatat 28949
                 .         .         .         .         .
ST21S   151 ATCATGAAAGGCTGGCTTTTTCTTGTTATCGCAATAGTTGGCGAAGTAAT 200
            ||||||||||||||||||||||||||||||||||||||||||||||||||
SGI1  28950 atcatgaaaggctggctttttcttgttatcgcaatagttggcgaagtaat 28999
```

```
                       .         .         .         .         .
ST21S    201 CGCAACATCCGCATTAAAATCTAGCGAGGGCTTTACTAAGCTTGCCCCTT 250
             ||||||||||||||||||||||||||||||||||||||||||||||||||
SGI1   29000 cgcaacatccgcattaaaatctagcgagggctttactaagcttgcccctt 29049
                       .         .         .         .         .
ST21S    251 CCGCCGTTGTCATAATCGGTTATGGCATCGCATTTTATTTTCTTTCTCTG 300
             ||||||||||||||||||||||||||||||||||||||||||||||||||
SGI1   29050 ccgccgttgtcataatcggttatggcatcgcattttattttctttctctg 29099
                       .         .         .         .         .
ST21S    301 GTTCTGAAATCCATCCCTGTCGGTGTTGCTTATGCAGTCTGGTCGGGACT 350
             ||||||||||||||||||||||||||||||||||||||||||||||||||
SGI1   29100 gttctgaaatccatccctgtcggtgttgcttatgcagtctggtcgggact 29149
                       .         .         .         .         .
ST21S    351 CGGCGTCGTCATAATTACAGCCATTGCCTGGTTGCTTCATGGGCAAAAGC 400
             ||||||||||||||||||||||||||||||||||||||||||||||||||
SGI1   29150 cggcgtcgtcataattacagccattgcctggttgcttcatgggcaaaagc 29199
                       .         .         .         .         .
ST21S    401 TTGATGCGTGGGGCTTTGTAGGTATGGGGCTCATAATTGCTGCCTTTTTG 450
             ||||||||||||||||||||||||||||||||||||||||||||||||||
SGI1   29200 ttgatgcgtggggctttgtaggtatggggctcataattgctgcctttttg 29249
                       .         .         .         .         .
ST21S    451 CTCGCCCGATCCCCATCGTGGAAGTCGCTGCGGAGGCCGACGCCATGGTG 500
             ||||||||||||||||||||||||||||||||||||||||||||||||||
SGI1   29250 ctcgcccgatccccatcgtggaagtcgctgcggaggccgacgccatggtg 29299
                       .         .         .         .         .
ST21S    501 ACGGTGTTCGGCATTCTGAATCTCACCGAGGACTCCTTCTTCGATGAGAG 550
             ||||||||||||||||||||||||||||||||||||||||||||||||||
SGI1   29300 acggtgttcggcattctgaatctcaccgaggactccttcttcgatgagag 29349
                       .         .         .         .         .
ST21S    551 CCGGCGGCTAGACCCCGCCGGCGCTGTCACCGCGGCGATCGAAATGCTGC 600
             ||||||||||||||||||||||||||||||||||||||||||||||||||
SGI1   29350 ccggcggctagaccccgccggcgctgtcaccgcggcgatcgaaatgctgc 29399
                       .         .         .         .         .
ST21S    601 GAGTCGGATCAGACGTCGTGGATGTCGGACCGGCCGCCAGCCATCCGGAC 650
             ||||||||||||||||||||||||||||||||||||||||||||||||||
SGI1   29400 gagtcggatcagacgtcgtggatgtcggaccggccgccagccatccggac 29449
                       .         .         .         .         .
ST21S    651 GCGAGGCCTGTATCGCCGGCCGATGAGATCAGACGTATTGCGCCGCTCTT 700
             ||||||||||||||||||||||||||||||||||||||||||||||||||
SGI1   29450 gcgaggcctgtatcgccggccgatgagatcagacgtattgcgccgctctt 29499
                      .         .         .         .         .
ST21S    701 AGACGCCCTGTCCGATCAGATGCACCGTGTTTCAATCGACAGCTTCCAAC 750
             ||||||||||||||||||||||||||||||||||||||||||||||||||
SGI1   29500 agacgccctgtccgatcagatgcaccgtgtttcaatcgacagcttccaac 29549
                       .         .         .         .         .
ST21S    751 CGGAAACCCAGCGCTATGCGCTCAAGCGCGGCGTGGGCTACCTGAACGAT 800
             ||||||||||||||||||||||||||||||||||||||||||||||||||
SGI1   29550 cggaaacccagcgctatgcgctcaagcgcggcgtgggctacctgaacgat 29599
                       .         .         .         .         .
ST21S    801 ATCCAAGGATTTCCTGACCCTGCGCTCTATCCCGATATTGCTGAGGCGGA 850
             ||||||||||||||||||||||||||||||||||||||||||||||||||
SGI1   29600 atccaaggatttcctgaccctgcgctctatcccgatattgctgaggcgga 29649
                       .         .         .         .         .
ST21S    851 CTGCAGGCTGGTGGTTATGCACTCAGCGCAGCGGGATGGCATCGCCACCC 900
             ||||||||||||||||||||||||||||||||||||||||||||||||||
SGI1   29650 ctgcaggctggtggttatgcactcagcgcagcgggatggcatcgccaccc 29699
                       .         .         .         .         .
ST21S    901 GCACCGGTCACCTTCGACCCGAAGACGCGCTCGACGAGATTGTGCGGTTC 950
             ||||||||||||||||||||||||||||||||||||||||||||||||||
SGI1   29700 gcaccggtcaccttcgacccgaagacgcgctcgacgagattgtgcggttc 29749
                       .         .         .         .         .
ST21S    951 TTCGAGGCGCGGGTTTCCGCCTTGCGACGGAGCGGGGTCGCTGCCGACCG 1000
             ||||||||||||||||||||||||||||||||||||||||||||||||||
SGI1   29750 ttcgaggcgcgggtttccgccttgcgacggagcggggtcgctgccgaccg 29799
```

```
                             .         .         .         .         .
ST21S  1001 GCTCATCCTCGATCCGGGGATGGGATTTTTCTTGAGCCCCGCACCGGAAA 1050
            |||||||||||||||||||||||        |||  ||  |
SGI1  29800 gctcatcctcgatccggggatggaccggcgattggtccatggcgaaacgg 29849

...
...

                             .         .         .         .         .
ST21S   990 GCTGCCGACCGGCTCATCCTCGATCCGGGGATGGGATTTTTCTTGAGCCC 1039
            ||||||||||||||||||||||||||||||||||||||||||||||||||
SGI1  39249 gctgccgaccggctcatcctcgatccggggatgggattttcttgagccc 39298
                             .         .         .         .         .
ST21S  1040 CGCACCGGAAACATCGCTGCACGTGCTGTCGAACCTTCAAAAGCTGAAGT 1089
            ||||||||||||||||||||||||||||||||||||||||||||||||||
SGI1  39299 cgcaccggaaacatcgctgcacgtgctgtcgaaccttcaaaagctgaagt 39348
                             .         .         .         .         .
ST21S  1090 CGGCGTTGGGGCTTCCGCTA.............................. 1109
            ||||||||||||||||||||
SGI1  39349 cggcgttggggcttccgctattggtctcggtgtcgcggaaatccttcttg 39398
```
