# Supplementary figures and images for: Stability, Entrapment and Variant Formation of Salmonella Genomic Island 1
Source: PLoS One. 2012 Feb 23;7(2):e32497. doi: 10.1371/journal.pone.0032497 (PMC3285670; doi:10.1371/journal.pone.0032497)

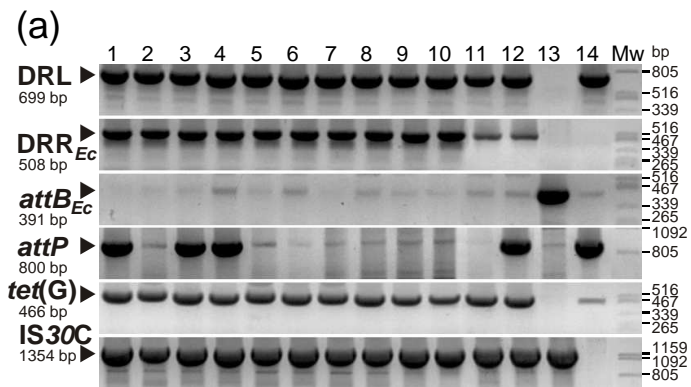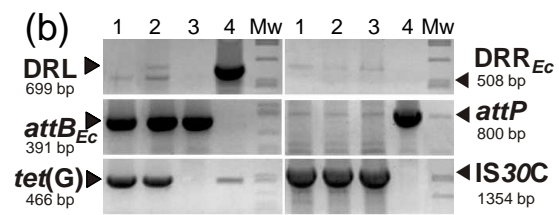

Supplement: Figure S2 — PCR analysis of the SGI1 transconjugant E. coli TG90Nal strains. A. Lanes 1–12 show the PCR amplicons from the total DNA of 12 randomly chosen NalRStrR TG90Nal transconjugant colonies, where the donor strains (all harbouring R55) were ST1134, ST1233, ST1288, ST1289, ST1321, ST1323, ST1367, ST1369, ST1373, ST1375, ST1772 or ST1773, respectively. Lane 13: TG90Nal recipient, Lane 14: ST1773/R55 donor. Primers were used as follows: DRL – attsgi1for-LJ2, DRREc – RJ2-attsgi1rev, attBEc – attsgi1for-attsgi1rev, attP – LJ2-RJ2, tet(G) – tetGfor-tetGrev, IS30C – IS30Cfor-IS30Crev. Positive signals for the DRL, DRREc, and tet(G) prove the presence of SGI1, while bands for DRREc and IS30C (specific for the chromosomal copy of E. coli IS element IS30, IS30C [1]) show that the sample colonies were E. coli. The faint positive signal for attBEc probably came from the attB site left behind SGI1 by its spontaneous excision as observed with the original Salmonella strains (see Fig. 1b). The expected fragment sizes are indicated. B. PCR tests for two representative NalRStrR TG90Nal transconjugant colonies, where the insertion occurred outside of the primary attB site. Lanes 1–2: two transconjugant colonies, where the donor strains were ST1289 and ST1773, respectively. Lanes 3–4 are TG90Nal recipient and ST1773/R55 donor, respectively. Reference: 1. Umeda M, Ohtsubo E (1990) Mapping of insertion element IS30 in the Escherichia coli K12 chromosome. Mol Gen Genet 222: 317–322. (PDF) [file pone.0032497.s002.pdf]
