# Supplementary material for: Stability, Entrapment and Variant Formation of Salmonella Genomic Island 1
Source: PLoS One. 2012 Feb 23;7(2):e32497. doi: 10.1371/journal.pone.0032497 (PMC3285670; doi:10.1371/journal.pone.0032497)
Supplement: Table S1 — Oligonucleotide primers used for PCR. (PDF) [file pone.0032497.s003.pdf]

Primers	*Mark on Figure 2*	*Sequence (5'-3')*	*References*
attsgi1for	-	gctctagagcggccgcatggaaggcggcttcctggc	This work
attsgi1rev	-	gctctagagcggccgcaaatgtgaatcgaatcacaatcg	This work
U7-L12	-	acaccttgagcagggcaaag	[1]
C9-L1[a]	-	gctctagagcggccgctcccctggaaattgcatag	This work
C9-L2	-	agcaagtgtgcgtaatttgg	[1]
sgi1Adelfor	*f*	cagaagctgggcgaacaaac	This work
sgi1Adelrev	*g*	gttcaacttgctgaaactttgaac	This work
sgi1Sdelfor	*d*	catcaagtcagttggtaaatgatg	This work
sgi1Sdelrev	*e*	ccaagaaggatttccgcgac	This work
sgi1seqfor	*a*	gagatcatctgcaggtgactgtaatc	This work
sgi1seqrev	*b*	aactgcagtggttgggtatacttcagc	This work
sgi1seqrev2	*c*	ttctgcagatgatcggacatagcgtt	This work
LJ2	-	agctgcagcggccgcaagttttactctgtcttccag	This work
RJ2	-	agctgcagcggccgctcgaagaggtagagcag	This work
tetGfor	-	gctcggtggtatctctgctc	[2]
tetGrev	-	agcaacagaatcgggaacac	[2]
flofor	*h*	aactgcaggccggtgcggagatggccg	This work
florev	*i*	aatctagatggtttcgagcggctgcgttc	This work
16Sfor	-	gagtttgatcctggctcag	[3]
16Srev	-	agaaaggaggtgatccagcc	[3]
IS30Cfor	-	agaattcttacttatgctgattccggtgcg	[3]
IS30Crev	-	ctcaagcttgatcgtgccactatttatacctg	[3]

[a] The original primer published by [1] has been modified by adding cleavage sites of *Xba*I and *Not*I to the 5' end.

References

1. Boyd DA, Peters GA, Ng L, Mulvey MR (2000) Partial characterization of a genomic island associated with the multidrug resistance region of *Salmonella enterica* Typhimurium DT104. FEMS Microbiol Lett 189: 285-291.
2. Ng LK, Martin I, Alfa M, Mulvey M (2001) Multiplex PCR for the detection of tetracycline resistant genes. Mol Cell Probes 15: 209-215.
3. Kiss J, Olasz F (1999) Formation and transposition of the covalently closed IS*30* circle: the relation between tandem dimers and monomeric circles. Mol Microbiol 34: 37-52.
